# Monthly Record of Deaths in the City of Buffalo

**Published:** 1857-10

**Authors:** C. L. Dayton

**Affiliations:** Health Physician


ART. VI.— *Report of Mortality in Buffalo for the month of Aug.*, 1857. By C. L. DAYTON, M. D., Health Physician.

| DISEASES. | No. | Males. | Females. |
|---|---|---|---|
| Accident, | 2 | 2 | |
| Asphyxia Immersorum, | 4 | 4 | |
| Asthma, | 1 | 1 | |
| Apoplexia, | 1 | | 1 |
| Cholera Morbus, | 1 | 1 | |
| Croup, | 2 | | 2 |
| Cholera Infantum, | 39 | 27 | 12 |
| " Morbus, | 6 | 3 | 3 |
| Cancrum Oris, | 2 | 1 | 1 |
| Carcinoma, | 2 | | 2 |
| Diarrhœa, | 31 | 16 | 15 |
| Dysenteria, | 12 | 5 | 7 |
| Dentitio, | 7 | 5 | 2 |
| Debilitas Senilis, | 6 | 4 | 2 |
| Erysipelas, | 1 | 1 | |
| Epilepsy, | 1 | 1 | |
| Eclampsia, | 20 | 7 | 13 |
| Febris Typhoid, | 1 | | 1 |
| " Scarlatina, | 4 | 1 | 3 |
| " Remittent, | 1 | 1 | |
| Gastritis, | 1 | 1 | |
| Hæmorrhagia Renalis, | 1 | 1 | |
| Hæmoptisis, | 1 | 1 | |
| Hemiplegia, | 1 | 1 | |
| Hydrocephalus, | 4 | 1 | 2 |
| Hydrops, | 5 | 1 | 4 |
| Hyperæmia Cerebralis, | 4 | 3 | 1 |

### REGISTER OF MORTALITY—Continued.

| DISEASES. | No. | Males. | Females. |
|---|---|---|---|
| Hyperæmia Pulmonalis, | 2 | 1 | 1 |
| Ignotus, | 28 | 17 | 11 |
| Intemperance, | 2 | 2 | |
| Meningitis, Cerebro Spinal, | 2 | 1 | 1 |
| Marasmus, | 15 | 10 | 5 |
| Pneumonitis, | 5 | 2 | 3 |
| Pertussis, | 3 | 2 | 1 |
| Premature Birth, | 2 | 2 | |
| Peritonitis, | 2 | 2 | |
| Rubeola, | 9 | 2 | 7 |
| Scrophula, | 2 | 1 | 1 |
| Syphilis, | 1 | | |
| Still-born, | 6 | 2 | 3 |
| Tuberculosis Pulmonalis, | 16 | 7 | 8 |
| Variola, | 1 | | 1 |
| Total, | 257 | | |

### SEXES.

| | |
|---|---|
| Males, | 140 |
| Females, | 113 |
| Sex not given, | 4 |
| Total, | 257 |

### AGES.

| | | | |
|---|---|---|---|
| Still-born, | 6 | Between 20 years and 30 years, | 13 |
| 1 day, | 1 | " 30 " " 40 " | 14 |
| 1 day and 30 days, | 25 | " 40 " " 50 " | 6 |
| Between 1 month and 6 months, | 55 | " 50 " " 60 " | 10 |
| " 6 months and 12 " | 52 | " 60 " " 70 " | 9 |
| " 1 year " 3 years, | 40 | " 70 " " 80 " | 2 |
| " 3 " " 5 " | 8 | " 80 " " 90 " | 1 |
| " 5 " " 10 " | 7 | " 90 " " 100 " | 0 |
| " 10 " " 20 " | 7 | " 100 " | 0 |
| Total number under 10 years, | 194 | Total number over 10 years, | 62 |
| Ages not given, | 1 | Total, | 257 |

### NATIVITIES.

| | | | |
|---|---|---|---|
| American, (white,) | 143 | Prussian, | 0 |
| " (colored,) | 3 | Welsh, | 0 |
| German, | 57 | French, | 0 |
| Irish, | 25 | Scotch, | 1 |
| English, | 5 | Holland, | 1 |
| Canadian, | 0 | Country not given, | 22 |
| | | Total, | 257 |

